# Toward an Understanding of the Lack of Transmission of Facts About Human Papillomavirus: Qualitative Case Study

**DOI:** 10.2196/64183

**Published:** 2025-08-15

**Authors:** Hind Bitar, Sarah Alismail

**Affiliations:** 1 King Abdulaziz University Jeddah Saudi Arabia; 2 Council of Health Insurance Riyadh Saudi Arabia

**Keywords:** human papillomavirus, HPV, information transmission, lack of knowledge, cervical cancer, health knowledge

## Abstract

**Background:**

Human papillomavirus (HPV) is the primary cause of cervical cancer, a largely preventable disease. Although extensive information about HPV is available and could help women prevent infection, a widespread lack of knowledge transmission hinders many women in Saudi Arabia from taking necessary preventive steps. Previous studies have reported low levels of HPV awareness among women in Saudi Arabia, highlighting the importance of understanding the barriers to effective information dissemination. Identifying the factors that influence the transmission of HPV-related knowledge is essential for designing targeted and impactful public health interventions.

**Objective:**

This study aimed to explore the factors that either block or facilitate the transmission of HPV-related facts among women in Saudi Arabia, using the HPV facts transmission model as a theoretical framework.

**Methods:**

A qualitative case study design was used, involving semistructured interviews with 20 women in Saudi Arabia aged 23 to 42 years. Participants were recruited using convenience and snowball sampling. The data were analyzed using pattern matching to assess how participant responses aligned with 11 predefined propositions from the HPV facts transmission model, which integrates individual and social influences on health information–seeking behavior.

**Results:**

Of the 11 propositions, 8 (73%) were supported by the data. Five were individual-level factors (personal need to learn, stigma, language barriers, technology use, and individual qualities), while 3 were social-level factors (social promotion, social support, and cultural norms). These factors were classified as barriers, resources, or both, depending on their influence on women’s intention to seek HPV-related knowledge. For instance, personal motivation, curiosity, and digital access facilitated knowledge acquisition, while stigma, limited Arabic-language resources, and conservative social norms served as major deterrents. Three propositions (social structure, suppression structure, and interaction or collaboration) did not align with participant experiences and were excluded from the final model.

**Conclusions:**

Understanding these barriers and resources is essential for developing targeted interventions to improve HPV knowledge dissemination. Strategies should include culturally appropriate awareness campaigns, accessible Arabic-language educational materials, and the integration of digital tools to encourage confidential learning. Addressing stigma through community engagement and structured education programs can further enhance HPV fact transmission, ultimately supporting informed decision-making and preventive health behaviors among women in Saudi Arabia.

## Introduction

Cervical cancer is the fourth most common cancer among women worldwide [1] and the eighth most common among women Saudi in Arabia aged 14 to 44 years [2]. In Saudi Arabia, the age-standardized rate of cervical cancer is relatively low at 2.2 per 100,000, yet the disease carries a 35% fatality rate [2]. Notably, >99% of cervical cancer cases are attributed to human papillomavirus (HPV) infections [3], making HPV knowledge a critical component of prevention. Despite the availability of information about HPV, such as that provided by authoritative sources such as the Centers for Disease Control and Prevention website [4], many women remain unaware of the health consequences of HPV [5]. For instance, a study among female university students in Saudi Arabia revealed that 95% demonstrated poor knowledge of cervical cancer and its link to HPV [6], while another study in Jazan City reported a mean knowledge score of only 1.99 out of 10 [7].

This persistent knowledge gap is not due to an absence of information but rather reflects barriers to effective information seeking and transmission. Factors such as social stigma surrounding sexually transmitted diseases (STDs), including HPV, constrain women’s willingness and ability to search for and internalize sexual health knowledge [8]. Poor awareness has been associated with delayed health care–seeking behaviors for STDs [9], further compounding public health challenges. For instance, one national survey of 755 women in Saudi Arabia found that only about 21% of them showed adequate knowledge about cervical cancer and HPV [10].

**Figure 1:**
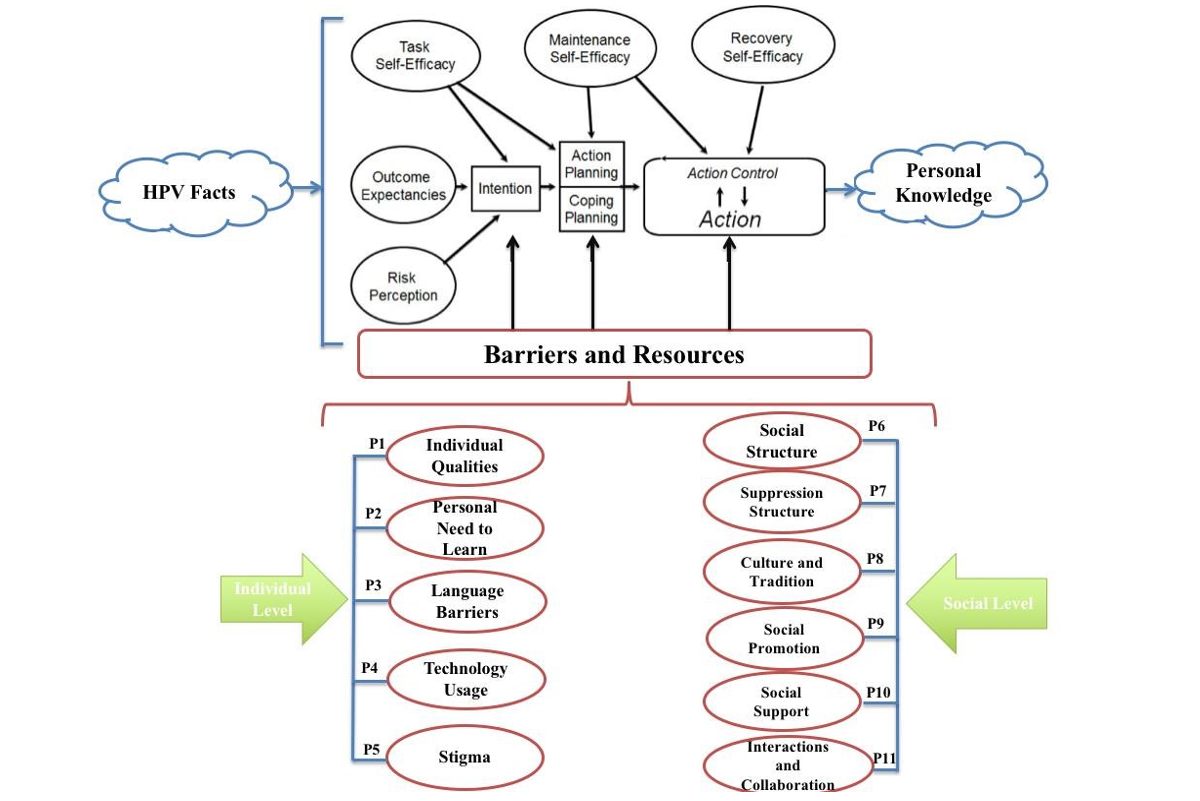
HPV Facts Transmission Model, including barriers and resources propositions. HPV: human papillomavirus.

To address this gap, we previously developed the HPV facts transmission model (Figure 1 [5]), a conceptual framework grounded in the health action process approach [11], which categorizes the barriers and resources influencing women’s access to, understanding of, and engagement with HPV-related information. The model emphasizes individual and social factors across preintentional (motivation), intentional (planning), and action phases. It proposes that motivation to seek knowledge is shaped by perceived risk, outcome expectancy, and self-efficacy. Once motivated, women enter an action planning phase, determining how and when to acquire information. This process culminates in the actual seeking and internalizing of HPV facts—what the model refers to as action and action control [5].

In this study, we build upon our previous work by empirically testing the motivation phase of the model through a qualitative investigation. Specifically, we explore how the model’s barrier and resource propositions influence women’s intentions to seek and acquire HPV-related knowledge. By doing so, we aim to validate the relevance of the HPV facts transmission model in real-world settings and to inform culturally sensitive strategies to enhance HPV awareness and education.

## Methods

### Overview

In our previous work, we developed and proposed the HPV facts transmission model [5] to explore the mechanisms influencing the dissemination of HPV-related information. This study builds upon that foundation by qualitatively examining the factors identified in the model, with a specific focus on the intention to seek and acquire knowledge about HPV. The HPV facts transmission model suggests that various factors can either facilitate or hinder the transmission of HPV-related information in Saudi Arabia, as outlined earlier in this paper. To investigate these factors, we use a qualitative case study design [12], which includes the following elements: questions and propositions, units of analysis, logic to link data to propositions, and interpretation of findings. This paper adheres to the COREQ (Consolidated Criteria for Reporting Qualitative Research) checklist, ensuring comprehensive and transparent reporting of qualitative methodologies (Multimedia Appendix 1 [13]). The researchers, all female, have extensive expertise in qualitative studies, which informed the design, execution, and analysis of this research.

### Ethical Considerations

This study was reviewed and approved by the institutional review board (IRB) of Claremont Graduate University (3101). Ethics approval was obtained to ensure compliance with human participant research guidelines and to protect their rights and well-being throughout the research process. Before participation, all participants provided written informed consent after being informed about the study’s objectives, procedures, and their rights, including the right to withdraw at any time without consequences. Given the sensitivity of the topic, verbal consent was also reaffirmed before each interview. To protect participant confidentiality, no personally identifiable information was collected. Participants were assigned unique codes (eg, P1 and P2) to anonymize their responses, and all data were securely stored and accessible only to the research team. In addition, interviews were not audio-recorded to allow participants to speak freely, with detailed notes taken instead. Participants were not financially compensated for their involvement to minimize potential coercion and ensure unbiased participation.

### Question and Propositions

This study aimed to answer the following question: What factors influence the intention to transmit HPV-related facts? As part of our previously developed HPV facts transmission model (illustrated in Figure 1), we identified 11 propositions related to factors that may either block or promote the transmission of HPV facts to women in Saudi Arabia. These propositions were originally conceptualized in our prior work based on theoretical frameworks and background literature. In this qualitative study, we empirically examined these propositions to determine their relevance and assess their role in influencing HPV knowledge–seeking behaviors.

The propositions for the *individual level* are as follows:

- *Individual qualities* affect the intention to transmit HPV facts.
- *Personal need to learn* affects the intention to transmit HPV facts.
- *Language barriers* affect the intention to transmit HPV facts.
- *Technology use* affects the intention to transmit HPV facts.
- *Stigma* affects the intention to transmit HPV facts.

The propositions for the *social level* are as follows:

- *Social structure* affects the intention to transmit HPV facts.
- *Suppression structure* affects the intention to transmit HPV facts.
- *Culture and tradition* affect the intention to transmit HPV facts.
- *Social promotion* affects the intention to transmit HPV facts.
- *Social support* affects the intention to transmit HPV facts.
- *Interaction and collaboration* affect the intention to transmit HPV facts.

### Data Collection

Semistructured interviews were used as a primary data collection technique. Interviews were conducted between April and June 2017. Questions about the 11 propositions were developed (Figure 1). When possible, questions were based on existing instruments. When not possible, background literature was searched to create questions. For example, the proposition *personal need to learn*—defined as the willingness to accept new knowledge [14]—was connected to the research objective, and the following interview question was generated: “If you know the health risks for any STDs such as HPV, are you intending to search for and learn about HPV? Why?” This example illustrates how each proposition informed the specific research questions used during data collection (Multimedia Appendix 2). Table 1 summarizes the methods used in developing the interview questions.

**Table 1:** Methods used in instrument development.

Propositions			Methods		References
**Individual level**					
	Individual qualities	Existing validated instruments (Curiosity and Exploration Inventory) and background literature		[3,15-18]	
	Personal need to learn	Background literature		[14]	
	Language barriers	Background literature		[19]	
	Technology use	Background literature		[19-21]	
	Stigma	Background literature		[22]	
**Social level**					
	Social structure	Background literature		[23]	
	Suppression structure	Background literature		[24]	
	Cultural and tradition	Background literature		[25]	
	Social promotion	Background literature		[26,27]	
	Social support	Existing validated instrument (Social Support Questionnaire-Short Form)		[28]	
	Interactions and collaboration	Existing validated instrument: the Interactions and Collaboration section from the Online Student Connectedness Survey		[29]	

All interviews were conducted in Arabic by the corresponding author, who was a doctoral student at the time of the study. At the time of the study, no prior relationship had been established between the researcher and the participants to minimize bias and ensure objectivity. Participants were informed about the general purpose of the study but were not given detailed insights into the researcher’s personal goals or assumptions to prevent response bias. To further minimize bias, the interviewer remained aware of potential assumptions throughout the research process, particularly regarding knowledge transmission and health information–seeking behaviors. The researcher team’s interest in the topic stemmed from prior work on HPV information dissemination and a commitment to understanding the barriers and facilitators influencing women’s access to HPV-related knowledge.

All interviews were conducted via phone calls, each lasting between 20 and 60 minutes. Interviews were not audio-recorded due to the sensitivity of the topic, allowing participants to speak freely. Instead, detailed field notes were taken during and immediately after each interview, capturing participant responses, key themes, and nonverbal cues. To ensure linguistic and cultural accuracy, the interview questions were developed as follows: (1) the first author wrote the questions in English and then translated them into Arabic; (2) an independent Arabic expert checked them for grammar and clarity; (3) a third bilingual person reviewed the questions in both languages; and (4) finally, the first author reviewed all questions once more.

### Sample

All participants were women in Saudi Arabia aged ≥20 years, recruited from the community. The age criterion was selected based on prior research indicating that HPV infrequently affects women aged <20 years [30]. Participants were recruited using convenience and snowball sampling, a widely used approach for sensitive research topics [31], after obtaining IRB approval. Initial participants were approached through personal and professional networks via WhatsApp, and they subsequently referred other eligible participants. Study invitations containing a brief description of the research purpose, eligibility criteria, and contact information were circulated through existing WhatsApp groups and direct messages within educational, health, and professional circles. Interested women were asked to privately respond directly via WhatsApp or email to express their interest in participating. Those who responded were subsequently provided with detailed study information and an explanation of the informed consent process. Recruitment continued until data saturation was reached, ensuring diverse perspectives from women.

Before data collection, the first author pilot-tested the interview questions with 3 women in Saudi Arabia (aged 30-40 years) recruited via snowball sampling. Each participant was interviewed separately, and following each interview, questions were modified and calibrated to improve clarity. No interviews were repeated.

### Data Analysis: Pattern Matching

This study used pattern matching as its primary analytic technique. According to Bitektine [32], this method requires “a theoretical pattern of expected outcomes,” as well as “an observed pattern of effects,” and then making “an attempt to match the two.” The researchers used pattern matching to make sense of the participants’ perceptions and opinions regarding the lack of HPV information transmission [16].

The researchers examined the data to determine whether the propositions block or promote the transfer of HPV facts, focusing only on the intention mechanism. During the analysis, the definitions and explanations of each proposition were used to assess how well they matched participant responses. For example, the proposition concerning culture and tradition suggests that single (unmarried) women in Saudi Arabia are often more hesitant to discuss sexual health topics compared to married women. This aligns with findings by Al-Saggaf [33], who reported that searching for and discussing sexual health topics is culturally sensitive for unmarried women in Saudi Arabia. Throughout the analysis, we used this framework to determine whether the suggested proposition matched the participants’ responses. Analysis was conducted manually by the authors.

### Positionality of the Research Team

The research team consisted of female researchers with expertise in qualitative research and public health, providing a culturally informed perspective on HPV knowledge–seeking behaviors. The corresponding author, who conducted the interviews, had no prior relationship with the participants, ensuring minimal bias in data collection. To further enhance trustworthiness, reflexivity was maintained by critically examining how the researchers’ perspectives, cultural backgrounds, and assumptions might shape data interpretation. This helped ensure that the findings were grounded in participant experiences rather than researcher bias.

## Results

### HPV Facts Transmission Model Examination

A total of 20 women aged between 23 and 42 years were interviewed; of these, 13 (65%) were married and 7 (35%) were single women. The women were from different regions of Saudi Arabia: 15% (3/20) each from the East, South, Center, and North, and 40% (8/20) from the West. The interviews each lasted between 20 and 60 minutes.

**Figure 2:**
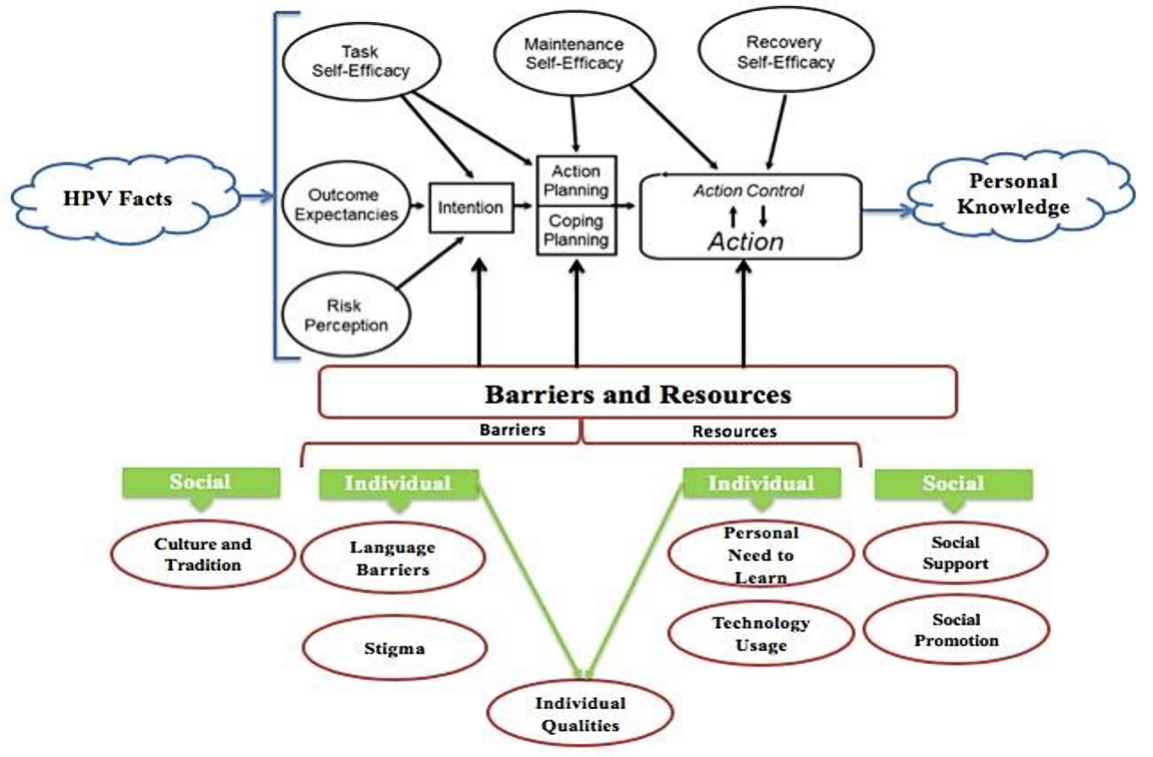
HPV Facts Transmission Model, including barriers and resources after analysis. HPV: human papillomavirus.

Of the 11 propositions, 8 (73%) were found to match the data well, while 3 (27%) did not. The *individual-level factors* include the *personal stigma, the need to learn, language barriers, technology use,* and *individual qualities*. The *social-level factors* include *social promotion, culture and tradition, and social support*. These 2 levels of factors can be arranged, as illustrated in Figure 2, into *barriers* and *resources*. Factors can be classified as either *barriers* or *resources*, based on how they impact women’s intention to search for and learn about HPV.

The subsequent subsections provide a detailed elaboration of the analysis for the matched and mismatched propositions.

### Matched Propositions: Each Factor’s Effects on Intention

The analyses show that, with respect to the effects of women’s searching and learning behaviors on their *personal knowledge*, 4 factors act as *barriers*, 5 factors act as *resources*, and 1 factor acts as both.

#### Culture and Tradition

Some participants asserted that *culture and tradition* can block the transfer of the facts about HPV. When the researchers asked the participants about their ability to talk about and discuss STDs such as HPV with others, a single woman answered as follows:

This topic is not easy to talk and discuss.

P6

In contrast, a married woman responded as follows:

I can talk, discuss with anyone, I do not care, but for single and divorced women, it is not appropriate.

P8

According to single women, talking about and discussing STDs such as HPV is not easy, as illustrated by P6, who claimed “I am shy.” All the married women interviewed for this study claimed not to care about what others may say about them. This reflects the findings by Al-Saggaf [33] regarding *culture and tradition* in Saudi Arabia. The predicted pattern matches the empirical pattern as follows:

- Empirical pattern—*culture and tradition*
  can deter the intention to transfer HPV facts.
- Predicted pattern—“*culture and tradition* affects the intention to transfer HPV facts.”

#### Language Barriers

*Language barriers* are a factor that can block the transfer of facts about HPV. As all the participants speak Arabic as their first—and in some cases only—language, they search for and learn about health information in Arabic. One example of this was a quote from a participant who stated the following:

I search and learn using the Arabic Language even though there are not a lot of materials in Arabic.

P10

The lack of educational materials about HPV available in Arabic can serve to block information seeking.

Some participants reported that if there were no Arabic materials available, they would stop their attempts to search for and learn about this type of information. A participant stated the following:

The only obstacle that I may face is the language barriers.

P4

Therefore, language is a *barrier* that can affect women’s acquisition of knowledge when searching for and learning about HPV. The predicted pattern matches the empirical pattern as follows:

- Empirical pattern—*language barriers* can deter the intention to transfer HPV facts.
- Predicted pattern—“*language barriers* affects the intention to transfer HPV facts.”

#### Stigma

*Stigma* results in bad feelings that affect personal and social relationships [25], which also can affect searching and learning behaviors regarding HPV. Most participants interviewed in this study search and learn secretly without telling anyone. A participant said the following:

If I could not get the information that I need regarding HPV, I may...ask someone whom I trust, but not a close one, such as my auntie because I am shy.

P6

Another participant responded the following:

...everything in my phone is hidden, where nobody can find it, not even my husband...In my society, if the husband knows that his wife is searching and learning about any sexual topics, he may divorce her.

P5

*Stigma* leads some women in Saudi Arabia to avoid talking about HPV with their close family members. Participants’ responses touched on their tendency to stop searching for and learning about HPV as well as avoid seeking answers from close family members due to fear of being marked with the stigma of HPV.

- Empirical pattern—stigma can deter the intention to transfer HPV facts.
- Predicted pattern—“stigma affects the intention to transfer HPV facts.”

#### Personal Need to Learn

Personal need to learn is a factor that can promote the transfer of HPV facts. Gaining the knowledge that a link exists between HPV and cervical cancer seems to lead women to ask questions about HPV infection. A participant stated the following:

Knowing the health risk of HPV really increased my intent to search and learn more about it; the nearer the distance between an individual and the risk, the more cautious an individual will be.

P4

A family history of cancer appears to intensify the influence of personal need on women’s health information–seeking and learning behaviors. A participant stated the following:

Since HPV causes cervical cancer, I may search and learn about this infection more because I have a family history of cancer.

P1

According to Duggan and Banwell [14], *personal need to learn* affects information dissemination or transmission. When women understand the serious health risks they face, they are more likely to seek out new knowledge to reduce those risks.

Therefore, *personal need to learn* also matches the predicted pattern:

- Empirical pattern—*personal need to learn* can promote and facilitate the intention to transfer HPV facts.
- Predicted pattern—“*personal need to learn* affects the intention to transfer HPV facts.”

#### Technology Use

The participants use technology in their daily lives, particularly smartphones. They use their smart devices to contact others, play games, search for information, and learn. One interviewee responded as follows:

Using technology is the easiest way to get information faster.

P3

Another interviewee said the following:

Technology is usually available to use anytime and anywhere.

P20

This factor is another resource at the individual level that participants use to obtain knowledge regarding any topics, including HPV. The women who were interviewed enjoy using technology because they can access information easily, quickly, anytime, anywhere, and anonymously. The empirical pattern matches the predicted pattern:

- Empirical pattern—*technology use* can promote and facilitate the intention to transfer HPV facts.
- Predicted pattern—“*technology use* affects the intention to transfer HPV facts.”

#### Social Support

*Social support* is defined as acquiring support (eg, help, information, or encouragement) from family, friends, and other sources [34]. Participants stated that if they were unable to obtain the information they needed through technology, they would seek it from trusted sources such as sisters, friends, or physicians. Thus, women in Saudi Arabia generally receive support from society.

This factor of social support was reflected in the participants’ responses. A participant reported the following:

I do not think that somebody will support me, but I do not care. I usually search secretly; nobody knows since I am using my laptop.

P6

Another participant stated the following:

No one will support me to search and learn about HPV, but I can ask my sisters, friends, and physicians if I need more information.

P1

These quotes suggest that, in the absence of support, the interviewed women may use technology to search for and learn about STDs. However, if they need additional information, they may seek assistance from others by asking specific questions.

The analysis results indicate that the *social support* factor promotes the intention to transfer HPV facts to women in Saudi Arabia who may benefit from such information, which thus matches the predicted pattern:

- Empirical pattern—*social support* can promote and facilitate the intention to transfer HPV facts.
- Predicted pattern—“*social support* affects the intention to transfer HPV facts.”

#### Social Promotion

All participants agreed that education programs may assist in increasing women’s *personal knowledge* of HPV infection. When the researchers asked about the effectiveness of education programs, a participant replied as follows:

Yes, I think having some education programs will help increase women’s awareness about STDs such as HPV and will educate them about it...this is considered as an easy way to get information.

P1

Another participant said the following:

Yes, this is a very good way to increase women’s sexual knowledge, but in an appropriate environment.

P20

These quotes suggest that education programs can provide women with easy access to accurate information, which is a supportive way to educate them on HPV.

As a result, *social promotion* through, for example, education programs can be considered a resource that promotes the transfer of HPV facts. This matched the predicted pattern:

- Empirical pattern—*social promotion* can promote and facilitate the intention to transfer HPV facts.
- Predicted pattern—“*social promotion* affects the intention to transfer HPV facts.”

#### Individual Qualities

*Individualqualities* can either facilitate or hinder the transfer of information about HPV, affecting women’s *personal knowledge*. *Individual qualities* consist of several characteristics, including curiosity, creativity, and willingness to change [4]. Moreover, the researchers observed that curiosity was the most influential factor affecting women’s knowledge. The more curious women are, the more willing they are to search for and learn about STDs. For example, when the researchers asked a participant about when she could search for and learn about STDs, she responded as follows:

I search and learn about some of the STDs such as HIV, but only if I am curious about it, and I will not search and learn about any topic that I am not interested in.

P9

Most participants agreed that if women are neither curious nor interested in HPV, they will neither search for nor learn about it. A participant said the following:

I do like to find new information and increase my knowledge if I think the topic is interesting.

P11

These 2 variables—women’s curiosity and their interest in the topic—are important elements that differ among women. These elements affect the searching and learning behaviors regarding STDs that enhance *personal knowledge*, which matches the predicted pattern:

- Empirical pattern—*individual qualities* can promote and facilitate the intention to transfer HPV facts.
- Predicted pattern—“*individual qualities* affects the intention to transfer HPV facts.”

### Mismatched Propositions

After analyzing the interview transcripts, the researchers removed 3 mismatched propositions, as described in the subsequent sections.

#### Social Structure

*Social structure* is defined as the social rules people follow in a society [26]. Participants agreed that searching for, learning about, talking about, and discussing HPV to increase women’s *personal knowledge* do not disobey society’s rules. A participant reported the following:

I do not think that searching and learning about STDs such as HPV is against the society’s rule...because knowing this information is similar to any other information that I may know, and it is not against the Islamic rules.

P1

Another participant reported the following:

I do not think that searching and learning about STDs such as HPV is against society’s rules; people now change. They want to educate themselves, especially if it is a disease.

P7

Saudi society seems to be changing, and the societal rules women in Saudi Arabia follow do not seem to have a strong effect on the transmission of HPV facts. The proposition concerning *social structure* does not manifest in promoting or blocking women in Saudi Arabia from searching for and learning about HPV. *Social structure* does not appear to be a factor in the HPV facts transmission model, as the interviewed Saudi women seem to engage in searching and learning activities with little concern for societal rules.

#### Suppression Structure

*Suppression structure* refers to how powerful individuals or groups may stop other individuals or groups from continuing their actions or activities [28]. The analysis result indicates that this proposition neither promotes nor blocks the transfer of the facts about HPV to women in Saudi Arabia who wish to enhance their knowledge.

When the researchers asked participants how they would react if someone in a position of power, such as a father or husband, forced them to stop their searching for and learning about HPV, a responded as follows:

No one can stop me because this is my personal choice...and this is my life, and I can do what I want.

P2

However, a single woman reported the following:

My mom may stop me, but not because searching and learning about HPV is against the society’s rules or anything else. She will stop me because I have an obsessive-compulsive disorder (OCD) and she is worried about me.

P6

These quotes suggest that *suppression structure* is likely not a factor that might affect the HPV facts transmission model.

#### Interactions and Collaboration

According to Barab et al [31], the proposition *interactions and collaboration* opens the opportunity to share experience and leverage a web-based interactive education program. Nonetheless, all participants classified HPV as a stigmatized disease, citing this stigma as the reason they prefer to search for and learn about this infection privately.

When the researchers asked them about their preference to search and learn about STDs, such as HPV, 1 participant stated the following:

I prefer to search and learn about HPV alone by myself because the way people think is different (not as me), and it is comfier to me (to search and learn by myself).

P1

This quote suggests that the interviewed women prefer to search for information about STDs, such as HPV, alone, as they feel more comfortable doing so.

Consequently, *interactions and collaboration* is likely not a factor that affects the HPV facts transmission model. This proposition will not create an opportunity to share experiences or facilitate online interactive discussions because HPV is a stigmatized disease.

## Discussion

### Principal Findings

This study examined the factors influencing HPV fact transmission among women in Saudi Arabia, using the HPV facts transmission model as a guiding framework. The initial model proposed 11 factors, but the qualitative analysis identified only 8 that shaped participants’ intention to seek and acquire HPV-related knowledge. These included 5 individual-level factors (personal need to learn, language barriers, stigma, technology use, and individual qualities) and 3 social-level factors (social promotion, social support, and cultural norms).

The findings reveal that personal need to learn and technology use were the strongest facilitators, enabling women to seek HPV-related information actively. In contrast, language barriers, stigma, and cultural norms emerged as key barriers, limiting knowledge transmission. Interestingly, social support played a dual role, acting as both a facilitator and a barrier, depending on the nature and source of support. These findings contribute to understanding the complex interplay between individual motivations, cultural norms, and technological influences in shaping women’s HPV knowledge–seeking behaviors.

### Comparison to Prior Work

The personal need to learn was identified as a key motivating factor, consistent with prior research on health information–seeking behaviors. Our findings align with those of Barbour et al [12], who argued that individuals actively seek health information when they recognize potential risks to their well-being. Similarly, the gratification theory by Wilson [35] states that “people are active seekers of information to gratify their needs.”

Technology use emerged as another important facilitator of HPV fact transmission. Participants emphasized the potential of digital platforms for overcoming barriers to information seeking, particularly through anonymized access to educational resources. This finding aligns with research by Hansen and Johnson [20], who introduced the concept of “veiled viral marketing”—a strategy that enables the dissemination of health information while preserving user anonymity [20]. Similarly, recent studies have highlighted the effectiveness of gamified mobile apps in enhancing users’ engagement with health information and empowering them to make informed health decisions [36,37].

Barriers to HPV knowledge transmission in this study also align with global research on stigma and language limitations. Participants reported a lack of Arabic-language educational materials, which restricted their ability to access reliable HPV-related information. Similar findings have been reported in Latinx communities in the United States, where language barriers negatively impact health care access and patient outcomes [38]. Our study suggests that localized, culturally relevant digital health interventions, such as Arabic-language eHealth platforms, could help mitigate these barriers and expand HPV awareness among women in Saudi Arabia.

Stigma was another major barrier, preventing women from openly discussing HPV or actively seeking information. This aligns with prior studies on STD-related stigma, which demonstrate that fear of judgment and cultural taboos affect women’s health care–seeking and decision-making behavior [39]. Similarly, our study found that women in Saudi Arabia tend to search for HPV information privately, avoiding discussions with family members or peers. Efforts to reduce health-related stigma in Saudi Arabia have been successful in other domains. For instance, the National Mental Health Program has effectively used community education campaigns to improve mental health literacy and normalize discussions about psychological well-being [40,41]. Similar awareness strategies could be applied to HPV education, focusing on reducing stigma, increasing public awareness, and normalizing sexual health discussions. However, as with mental health advocacy, HPV-related interventions will need to navigate cultural sensitivities carefully and ensure that educational campaigns respect social norms while promoting open dialogue.

Individual qualities are the final factor at the individual level that may promote or block women’s searching and learning behaviors to enhance their personal health knowledge. Among the various characteristic of this factor, curiosity emerged as particularly substantial. Participants expressed a greater inclination to search for and learn about HPV when they found the topic personally relevant or intriguing. This aligns with the Gratification Theory by Wilson [35], which suggests that individuals actively seek information to fulfill their personal knowledge needs. Wilson [35] further describes the concept of selective exposure, where individuals tend to seek out information that matches their interests and preexisting beliefs. This was evident in this study, where women who perceived HPV as a serious health risk were more likely to seek information, whereas those with less personal curiosity demonstrated lower engagement in knowledge acquisition.

At the social level, 3 factors—social promotion, social support, and cultural traditions—emerged as key influencers in HPV fact transmission. Social promotion, particularly in the form of educational programs, was identified as an effective method for enhancing HPV awareness among participants. Kirby et al [26] demonstrated that STD education programs positively influenced participants’ sexual health knowledge and behaviors, reinforcing the importance of structured, targeted awareness campaigns. However, some participants in this study emphasized that the effectiveness of social promotion depends on access to appropriate educational environments, indicating the need for well-designed interventions that are both culturally appropriate and widely accessible.

Social support was also found to be a major factor influencing HPV fact transmission, with trusted family members and friends playing a major role in facilitating or hindering knowledge sharing. The importance of social support in health education has been well documented. For example, Pratt’s study on hygiene education in 1905 found that socially supported individuals exhibited better health outcomes compared to those without support [42]. This aligns with the findings of this study that women who had access to supportive social networks were more likely to engage in HPV-related discussions and seek further knowledge. Conversely, participants who lacked support or feared judgment were less likely to openly discuss HPV or seek information.

In contrast, cultural traditions were identified as a barrier to HPV knowledge transmission, particularly for single women. Participants reported that searching for, discussing, talking about, and becoming educated on sexual topics are generally perceived as challenging tasks for single women, while these tasks typically do not bother married women. This finding aligns with the research conducted by Fageeh [43], which identified a lack of sexual health awareness as a primary factor contributing to STD transmission between couples in Saudi Arabia. The cultural discouragement of discussions surrounding sexual health continues to hinder information dissemination, suggesting the need for culturally sensitive awareness initiatives that respect social norms while promoting accurate health education.

In addition, 3 social-level propositions—social structure, suppression structure, and interactions or collaboration—were not supported by participant responses and were therefore eliminated from the final model. Participants largely agreed that learning about HPV does not violate societal norms, indicating that women in Saudi Arabia are increasingly educated and empowered to seek health information. For example, the campaign by Fageeh [43] on STD awareness was successfully launched in Saudi Arabia, suggesting that societal changes are making health education more accessible. If *social structure* affected the ability to disseminate HPV facts, then launching a campaign would have been impossible.

Finally, the proposition interactions and collaboration was eliminated due to mismatching. Although interactive health education programs have been effective in other contexts [44], participants in this study preferred private, self-directed learning rather than collaborative discussions about HPV. This highlights the importance of confidential educational approaches, such as digital health tools, to accommodate women’s preferences for privacy while seeking health information.

### Theoretical and Practical Contributions

This study makes significant contributions to both theory and practice by deepening our understanding of HPV knowledge transmission and offering insights for health interventions. Theoretically, this study provides an exemplar for the theory of the problem by Markus [41], which states that a research article should explain how and why a problem occurs, supported by qualitative data or other empirical evidence. By identifying the barriers and facilitators influencing HPV fact transmission, this study establishes a theory of the problem, offering a structured explanation of why HPV knowledge remains inadequately disseminated among women in Saudi Arabia. The refined HPV facts transmission model meets the standards for problem-driven theoretical contributions by Markus [41], as it is concrete, contextually specific, and focused on a real-world phenomenon. From a practical perspective, this research provides actionable insights for public health practitioners, educators, and policy makers. The study identifies 8 key factors that act as barriers, resources, or both in HPV fact transmission. These findings can inform the design of targeted interventions aimed at enhancing HPV awareness and education in Saudi Arabia.

Specifically, this study highlights the importance of the following:

- Leveraging digital health solutions such as mobile apps, social media campaigns, and online educational platforms to address language barriers and stigma while ensuring confidential access to HPV-related information
- Enhancing social support networks and encouraging trusted sources of information, such as health care providers, community leaders, and family networks, to facilitate accurate HPV knowledge sharing
- Implementing culturally sensitive educational programs and ensuring that awareness campaigns respect social norms while promoting factual, accessible health information

By validating and refining the HPV facts transmission model, this study provides a conceptual framework that can be used to inform public health initiatives, guide future research, and enhance HPV-related interventions. These findings emphasize the need for culturally appropriate, evidence-based strategies to improve HPV knowledge transmission, ultimately supporting cervical cancer prevention efforts in Saudi Arabia and similar contexts.

### Limitations and Future Directions

The study has some limitations that should be considered when interpreting the findings. First, it is important to note that the data were collected 8 years ago. Since then, the modes and speed of health information transmission, particularly digital platforms and social media, have evolved substantially. While the insights remain valuable for understanding cultural and structural influences on HPV-related information seeking, the findings should be interpreted within the temporal context of the study. Future research should examine how technological advances and shifts in public health communication strategies have reshaped information access and behaviors in more recent years. Second, participant personal experiences with HPV, such as having tested positive for the virus or having received the HPV vaccine, were not collected. These factors could have influenced participants’ decision to take part in the study as well as their motivation to seek information about HPV, potentially shaping the perspectives they shared during the interviews. Third, participants were not given the opportunity to review their interview transcripts or the results of the thematic analysis. Although reflexive and iterative coding processes were used to enhance analytical rigor, member checking might have strengthened the credibility and accuracy of the findings by allowing participants to clarify or expand on their perspectives. Fourth, as in all qualitative research, the potential for researcher bias exists and may influence the data collection, analysis, and interpretation. Although we used strategies such as peer debriefing and maintained detailed reflexive notes to enhance trustworthiness, complete objectivity in qualitative interpretation cannot be assumed. Fifth, the use of snowball and convenience sampling means that our findings are specific to the study participants and may not be transferable to the broader population. However, the aim was not to draw wider inferences but rather to gain a profound understanding of the factors influencing HPV facts transmission within the specific sample, and efforts were made to include women from various regions to capture diverse perspectives. Sixth, the voluntary nature of participation may have introduced selection bias. While we cannot eliminate selection bias entirely, we minimized its potential impact by inviting all interested women, regardless of their knowledge or opinions, to participate. Seventh, the focus was primarily on women’s marital status in relation to cultural influences, without fully exploring other potential cultural variations. Regional differences could significantly impact individuals’ experiences and behaviors related to sexual health education. To mitigate this, we made an effort to include women from different regions of Saudi Arabia in our sample. However, we acknowledge that a more in-depth exploration of other cultural factors, including regional nuances, would be necessary in future research to provide a more comprehensive understanding of how cultural context shapes HPV-related knowledge-seeking behavior across different parts of the country.

Despite these limitations, this study provides a valuable foundation for future research. Future studies could explore the development and testing of digital interventions, such as IT- or information system–based tools, to improve the accessibility and effectiveness of HPV information dissemination. In addition, incorporating other qualitative data-gathering techniques, such as focus groups, could provide richer insights into participants’ experiences and further inform intervention strategies. Future research could also explore the role of organizational social support in facilitating knowledge sharing about stigmatized health conditions, such as HPV. Assessing how organizations can provide support for individuals in seeking information and reducing stigma could offer valuable insights into the development of effective interventions. By understanding the factors that promote or block the transmission of HPV-related information among women in Saudi Arabia, strategies can be devised to enhance knowledge dissemination and support informed health-related decisions, ultimately contributing to improved women’s health.

### Conclusions

HPV is the primary cause of cervical cancer worldwide; however, despite the availability of factual information, many women remain uninformed or misinformed, limiting their ability to take preventative measures against infection. This study examined the factors that facilitate or hinder the transmission of HPV-related knowledge among women in Saudi Arabia, offering insights into the barriers and enablers that shape health information–seeking behaviors. The findings identified 8 key factors that impact HPV fact transmission, categorized into individual-level and social-level influences. The study’s findings underscore the importance of addressing these barriers through culturally appropriate interventions. Public health strategies should focus on leveraging digital health resources, fostering supportive social networks, and implementing targeted educational programs to improve HPV awareness. By addressing sociocultural constraints and structural limitations, these interventions can help enhance knowledge dissemination and promote informed decision-making among women in Saudi Arabia.

## Appendix

Multimedia Appendix 1 COREQ (Consolidated Criteria for Reporting Qualitative Studies) 32-item checklist. Developed from the study by Tong A et al [13].

Multimedia Appendix 2 Semistructured interview questions.

## Abbreviations

COREQ: Consolidated Criteria for Reporting Qualitative Research
HPV: human papillomavirus
IRB: institutional review board
STD: sexually transmitted disease

## Data Availability

The datasets generated or analyzed during this study are available from the corresponding author on reasonable request.
