# Supplementary material for: Toward an Understanding of the Lack of Transmission of Facts About Human Papillomavirus: Qualitative Case Study
Source: JMIR Cancer. 2025 Aug 15;11:e64183. doi: 10.2196/64183 (PMC12397709; doi:10.2196/64183)
Supplement: Multimedia Appendix 2 [file cancer_v11i1e64183_app2.docx]

**Appendix 2: Semi-structured Interview Questions**

**Proposition (1) *Individual Qualities*:**

- Do you know what HPV is? Explain.
- Do you know what causes cervical cancer? Explain.
- How have you previously gain knowledge/information regarding STDs such as HPV?
- Would you like to increase your knowledge regarding STDs such as HPV? If yes, please explain how or how would you gain/increase your health knowledge/information regarding STDs such as HPV? If your answer is no please explain ,why?
- Who could support you in increasing your knowledge regarding STDs such as HPV? Why?
- If you cannot get the information that you want regarding any of STDs such as HPV, what will you do?
- How would you describe yourself in seeking information about STDs such as HPV?
- If you are participating in an activity, do you usually keep track of time? Why?
- Do you like to look for new opportunities to increase your knowledge about STDs such as HPV? If your answer is yes, please explain how, if your answer is no, explain why.
- Do you like to probe deeply when you are learning new things? Why?
- Have you thought about a detailed plan how to search and learn about STDs such as HPV to increase your knowledge?

**Proposition (2) *Personal Need to Learn*:**

- If you know the health risks for any STDs such as HPV, are you intending to search and

learn about HPV? Why?

**Proposition (3) *Language Barriers*:**

- What is the primary language that you speak?
- If you intend to search and learn about STDs, such as HPV what is the language that you prefer to use during the searching and learning? Why?
- Do you speak another language?
  1. If yes, if you intend to search and learn about STDs such as HPV, would you also look using this language? Why?

**Proposition (4) *Technology Usage*:**

- If you intend to search and lean about STDs such as HPV, would you use technology to search and learn about HPV?
- If you intend to search and lean about STDs such as HPV, how do you use technology to search and learn about them?
- Why do you use technology to search and learn about STDs such as HPV?

**Proposition (5) *Stigma*:**

- Do you worry about what other people (family members, friends, etc....) would think of

you if they know that you are intending to search and learn about STDs, such as HPV?

Why?

- If you intend to search and learn about STDs, such as HPV’s definitions, symptoms, causes, etc., when would you stop seeking information? Why?

**The *Social Level* Factors:**

**Proposition (6) *Social Structure*:**

- If you intend to search and learn about STDs such as HPV, do you think that searching and learning about STDs such as HPV is against your society’s rules? Why?
- If you intend to search and learn about STDs such as HPV, does your status (single, married) affect your action and decision to search and learn? Why?

**Proposition (7) *Suppression Structure*:**

- If you intend to search and learn about STDs such as HPV, can your father, husband, mother, or anyone who has the power stop you? Why?

**Proposition (8) *Culture and Tradition*:**

- What words would you use to describe your own culture’s ideas regarding discussions about STDs such as HPV?
- If you intend to search and lean about STDs such as HPV, can you discuses and talk with friends, family, and relatives about STDs, such HPV? Explain.

**Proposition (9) *Social Promotion:***

- Could you explain what would motivate you to search and learn about STDs such as HPV?
- Do you agree with the following statement: “education program positively affected the sexual behaviors of participates”? Why?
- Do you think that education programs about STDs such as HPV could make women aware and encourage/motivate them to search and learn about HPV? Why?

**Proposition (10) *Social Support:***

- If you intend to search and learn to increase your knowledge/information about STDs such as HPV, whom will you trust? Why?
- If you intend to search and learn about STDs such as HPV and you need help, whom will you ask? Why?

**Proposition (11) *Interaction and Collaboration:***

- If you intend to search and learn to increase your knowledge/information about STDs such as HPV, do you collaborate while you are searching and learning? Why?
- If you intend to search and learn to increase your knowledge/information about STDs such as HPV, do you share the knowledge that you have regarding HPV to others? Why?
